# Learning stochastic object models from medical imaging measurements by use of advanced ambient generative adversarial networks

**DOI:** 10.1117/1.JMI.9.1.015503

**Published:** 2022-02-23

**Authors:** Weimin Zhou, Sayantan Bhadra, Frank J. Brooks, Hua Li, Mark A. Anastasio

**Affiliations:** a University of California Santa Barbara, Department of Psychological and Brain Sciences, Santa Barbara, California, United States; b Washington University in St. Louis, Department of Computer Science and Engineering, St. Louis, Missouri, United States; c University of Illinois at Urbana-Champaign, Department of Bioengineering, Urbana, Illinois, United States; d Washington University in St. Louis, Department of Radiation Oncology, St. Louis, Missouri, United States; e Cancer Center at Illinois, Urbana, Illinois, United States

**Keywords:** objective assessment of image quality, stochastic object models, generative adversarial networks

## Abstract

**Purpose:**

To objectively assess new medical imaging technologies via computer-simulations, it is important to account for the variability in the ensemble of objects to be imaged. This source of variability can be described by stochastic object models (SOMs). It is generally desirable to establish SOMs from experimental imaging measurements acquired by use of a well-characterized imaging system, but this task has remained challenging.

**Approach:**

A generative adversarial network (GAN)-based method that employs AmbientGANs with modern progressive or multiresolution training approaches is proposed. AmbientGANs established using the proposed training procedure are systematically validated in a controlled way using computer-simulated magnetic resonance imaging (MRI) data corresponding to a stylized imaging system. Emulated single-coil experimental MRI data are also employed to demonstrate the methods under less stylized conditions.

**Results:**

The proposed AmbientGAN method can generate clean images when the imaging measurements are contaminated by measurement noise. When the imaging measurement data are incomplete, the proposed AmbientGAN can reliably learn the distribution of the measurement components of the objects.

**Conclusions:**

Both visual examinations and quantitative analyses, including task-specific validations using the Hotelling observer, demonstrated that the proposed AmbientGAN method holds promise to establish realistic SOMs from imaging measurements.

## 1 Introduction

Computer simulation remains an important approach for the design and optimization of imaging systems. Such approaches can permit the exploration, refinement, and assessment of a variety of system designs that would be infeasible through experimental studies alone.^1^^–^^3^ In the field of medical imaging, it has been advocated that imaging systems and reconstruction algorithms should be assessed and optimized using objective measures of image quality (IQ) that quantify the performance of an observer at specific diagnostic tasks.^4^^–^^8^ To accomplish this, all sources of variability in the measured data should be accounted for. One important source of variability that can significantly limit observer performance is variation in the objects to be imaged.^9^ This source of variability can be described by stochastic object models (SOMs).^10^ A SOM is a generative model that can be employed to produce an ensemble of to-be-imaged objects that possess prescribed statistical properties.

Available SOMs include texture models of mammographic images with clustered lumpy backgrounds,^11^ simple lumpy background models,^9^ and more realistic anatomical phantoms that can be randomly perturbed.^12^ A variety of other computational phantoms,^12^^–^^19^ either voxelized or mathematical, have been proposed for medical imaging simulation, aiming to provide a practical solution to characterize object variability. However, the majority of these were established using image data corresponding to only a few subjects. Therefore, they may not accurately describe the statistical properties of the ensemble of objects that is relevant to an imaging system optimization task. A variety of anatomical shape models have also been proposed to describe both the common geometric features and the geometric variability among instances of the population for shape analysis applications.^20^^–^^27^ To date, these have not been systematically explored for the purpose of constructing SOMs that capture realistic anatomical variations for use in imaging system optimization.

To establish SOMs that capture realistic textures and anatomical variations, it is desirable to utilize experimental imaging data. By definition, however, SOMs should be independent of the imaging system, measurement noise, and any reconstruction method employed. In other words, they should provide an *in silico* representation of the ensemble of objects to be imaged and not estimates of them that would be indirectly measured or computed by imaging systems. To address this need, Kupinski et al.^10^ proposed an explicit generative model for describing object statistics that was trained using noisy imaging measurements and a computational model of a well-characterized imaging system.^10^ However, applications of this method have been limited to situations where the characteristic functional of the random object can be analytically determined,^28^ such as with lumpy and clustered lumpy object models.^11^^,^^29^ As such, there remains an important need to generalize the method.

Deep generative neural networks, such as generative adversarial networks (GANs),^30^ hold great potential for establishing SOMs that describe finite-dimensional approximations of objects. However, conventional GANs are typically trained using reconstructed images that are influenced by the effects of measurement noise and the reconstruction process. To circumvent this, an AmbientGAN has been proposed^31^ that augments a GAN with a measurement operator. This permits a generative model that describes object randomness to be learned from indirect and noisy measurements of the objects themselves. In a preliminary study, the AmbientGAN was explored for establishing SOMs from imaging measurements for use in optimizing imaging systems.^32^ However, similar to conventional GANs, the process of training AmbientGANs is inherently unstable. Moreover, the original AmbientGAN cannot immediately benefit from robust GAN training procedures, such as progressive growing,^33^ which limits its ability to synthesize high-dimensional images that depict accurate approximations of objects that are relevant to medical imaging studies.

In this work, modern multiresolution training approaches, such as employed in the progressive growing of GANs (ProGANs)^33^ and style-based GANs (StyGANs),^34^^,^^35^ are modified for use in establishing AmbientGANs with high-dimensional medical imaging measurements. The resulting models will be referred to as progressive growing AmbientGANs (ProAmGANs) and style-AmbientGANs (StyAmGANs). Numerical studies corresponding to a stylized imaging system are conducted to systematically investigate the proposed advanced AmbientGAN methods for establishing SOMs. The effects of noise levels and the imaging operator null space characteristics on model performance are assessed using both standard and objective measures. Emulated single-coil experimental magnetic resonance imaging (MRI) data are also employed to demonstrate the method under less stylized conditions.

The remainder of this paper is organized as follows. In Sec. 2, previous works on learning SOMs by employing characteristic functions (CFs) and AmbientGANs are summarized. The progressive growing training strategy for GANs is also reviewed. The proposed ProAmGAN and StyAmGAN for learning SOMs from noisy imaging measurements are described in Sec. 3. Sections 4 and 5 describe the numerical studies and results that demonstrate the ability of the advanced AmbientGANs to learn SOMs from noisy imaging measurements. Finally, a discussion and summary of the work is presented in Sec. 6.

## 2 Background

Object properties that are imaged by medical imaging systems are inherently described by continuous functions. However, it is common practice when performing computer-simulation studies of imaging systems to approximate the object by use of a finite-dimensional representation.^36^^,^^37^ In such cases, a discrete-to-discrete (D-D) description of a linear imaging system can be described as^7^

g=Hf+n, (1)

where $g\in R^{M}$ is a vector that describes the measured image data, $f\in R^{N}$ denotes the finite-dimensional representation of the object being imaged, $H\in R^{M\times N}$ denotes a D-D imaging operator $R^{N}\to R^{M}$ that maps an object in the Hilbert space U to the measured discrete data in the Hilbert space V, and the random vector $n\in R^{M}$ denotes the measurement noise. Below, the imaging process described in Eq. (1) is denoted as: $g=H_{n}(f)$. In this work, it will be assumed that the D-D imaging model is a sufficiently accurate representation of the true continuous-to-discrete (C-D) imaging model that describes a digital imaging system and the impact of model error will be neglected. Accordingly, as described below, the objective of this work will be to establish SOMs that describe the finite-dimensional vector f.

When optimizing imaging system performance using objective measures of IQ, all sources of randomness in g should be considered. In diagnostic imaging applications, object variability is an important factor that limits observer performance. In such applications, the object f should be described as a random vector that is characterized by a multivariate probability density function (PDF) p(f) that specifies the statistical properties of the ensemble of objects to be imaged.

Direct estimation of p(f) is rarely tractable in medical imaging applications due to the high dimensionality of f. To circumvent this difficulty, a parameterized generative model, referred to throughout this work as an SOM, can be introduced and established using an ensemble of experimental measurements. The generative model can be explicit or implicit. Explicit generative models seek to approximate p(f), or equivalently, its CF, from which samples f can subsequently be drawn. On the other hand, implicit generative models do not seek to estimate p(f) directly, but rather define a stochastic process that can draw samples from p(f) without having to explicitly specify it. Variational autoencoders and GANs are examples of explicit and implicit generative models, respectively, that have been actively explored.^38^ Two previous works that sought to learn SOMs from noisy and indirect imaging measurements using explicit and implicit generative models are presented below.

### 2.1 Establishing SOMs using Explicit Generative Modeling: Propagation of Characteristic Functionals

The first method to learn SOMs from imaging measurements was introduced by Kupinski et al.^10^ In that seminal work, a C-D imaging model was considered in which a function that describes the object is mapped to a finite-dimensional image vector g. For C-D operators, it has been demonstrated that the characteristic functional (CFl) describing the object can be readily related to the CF of the measured data vector g.^39^ This provides a relationship between the PDFs of the object and measured image data. In their method, an object that was parameterized by the vector Θ was considered and analytic expressions for the CFl were utilized. Subsequently, using the known imaging operator and noise model, the corresponding CF was computed. The vector Θ was estimated by minimizing the discrepancy between this model-based CF and an empirical estimate of the CF computed from an ensemble of noisy imaging measurements. From the estimated CFl, an ensemble of objects could be generated. This method was applied to establish SOMs where the CFl of the object can be analytically determined. Such cases include the lumpy object model^29^ and clustered lumpy object model.^11^ The applicability of the method to more complicated object models remains unexplored.

### 2.2 Establishing Finite-Dimensional SOMs by Use of Implicit Generative Modeling: GANs and AmbientGANs

GANs^30^^,^^40^^–^^49^ are implicit generative models that have been actively explored to learn the statistical properties of ensembles of images (i.e., finite-dimensional approximations of object properties) and generate new images that are consistent with them. A traditional GAN consists of two deep neural networks: a generator and a discriminator. The generator is jointly trained with the discriminator through an adversarial process. During its training process, the generator is trained to map random low-dimensional latent vectors to higher dimensional images that represent samples from the distribution of training images. The discriminator is trained to distinguish the generated, or synthesized, images from the actual training images. These are often referred to as the “fake” and “real” images in the GAN literature. Subsequent to training, the discriminator is discarded and the generator and associated latent vector probability distribution form as an implicit generative model that can sample from the data distribution to produce new images. However, images produced by imaging systems are contaminated by measurement noise and potentially an image reconstruction process. Therefore, GANs trained directly on images do not generally represent SOMs because they do not characterize object variability alone.

**Fig. 1:**
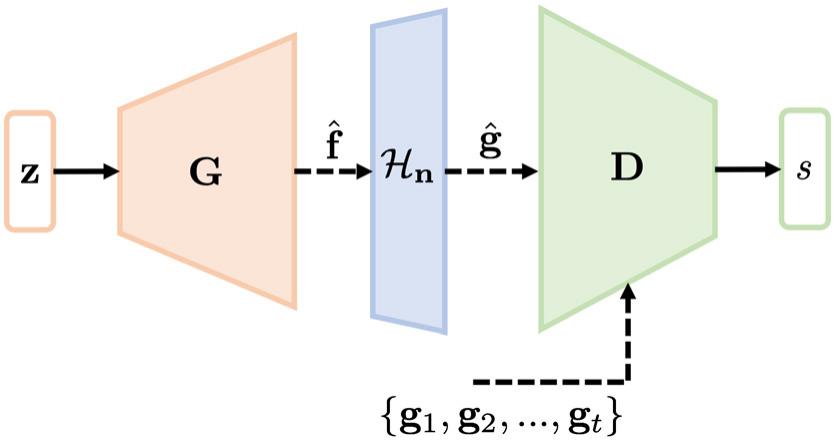
An illustration of the AmbientGAN architecture. The generator G is trained to generate objects, which are subsequently employed to simulate measurement data. The discriminator D is trained to distinguish “real” measurement data from the “fake” measurement data that are simulated by use of the generated objects.

An augmented GAN architecture named AmbientGAN has been proposed^31^ that enables learning an SOM that describes the statistical properties of finite-dimensional approximations of objects from noisy indirect measurements of the objects. As shown in Fig. 1, the AmbientGAN architecture incorporates the measurement operator $H_{n}$, defined in Eq. (1), into the traditional GAN framework. During the AmbientGAN training process, the generator is trained to map a random vector $z\in R^{k}$ described by a latent probability distribution to a generated object f^=G(z;ΘG), where $G:R^{k}\to R^{N}$ represents the generator network that is parameterized by a vector of trainable parameters $\Theta_{G}$. Subsequently, the corresponding simulated imaging measurements are computed as g^=Hn(f^). The discriminator neural network $D:R^{M}\to R$, which is parameterized by the vector $\Theta_{D}$, is trained to distinguish the real and simulated imaging measurements by mapping them to a real-valued scalar s. The adversarial training process can be represented by the following two-player minimax game:^30^

minΘG maxΘD V(D,G)=Eg∼p(g)[l(D(g;ΘD))]+Eg^∼p(g^)[l(1−D(g^;ΘD))], (2)

where l(·) represents a loss function. When the distribution of objects p(f) uniquely induces the distribution of imaging measurements p(g), i.e., when the imaging operator is injective, and the minimax game achieves the global optimum, the trained generator can be employed to produce object samples drawn from p(f).^30^^,^^31^

Zhou et al.^32^ demonstrated the ability of the AmbientGAN to learn a simple SOM corresponding to a lumpy object model that could be employed to produce small (64×64) object samples. However, adversarial training is known to be unstable and the use of AmbientGANs to establish realistic and large-scale SOMs has, to date, been limited.

### 2.3 Advanced GAN Training Strategies

A training strategy for GANs—progressive growing of GANs (ProGANs)—has been recently developed to improve the stability of the GAN training process^33^ and hence the ability to learn generators that sample from distributions of high-resolution images. GANs are conventionally trained directly on full size images through the entire training process. In contrast, ProGANs adopt a multiresolution approach to training. Initially, a generator and discriminator are trained using downsampled (low resolution) training images. During each subsequent training stage, higher resolution versions of the original training images are employed to train progressively deeper discriminators and generators, continuing until a final version of the generator is trained using the original high-resolution images. A similar progressive training procedure is employed in the StyleGAN framework.^34^ More recently, an advanced GAN training strategy—StyleGAN2—has been developed to further improve the IQ of the synthesized images.^35^ Although, StyleGAN2 does not employ the progressive growing strategy, the generator does make use of multiple scales of image generation via skip connections between lower resolution generated images to the final generated image.^35^ While these advanced training strategies have found widespread success on training GANs, they cannot be directly used to train AmbientGANs for establishing SOMs from medical imaging measurements. This is because these GAN training procedures and architectures are designed to train the generator that produces images in the same Hilbert space as the training images. However, medical imaging measurements g that are used as training data of AmbientGANs are typically indirect representations of to-be-imaged objects f and generally reside in a different Hilbert space than the generator-produced objects f^. For example, in MRI, the to-be-imaged objects reside in a real Hilbert space while the k-space measurements reside in a complex Hilbert space. A solution to this problem that enables the use of advanced GAN training methods for training AmbientGANs is described next.

## 3 Establishing SOMs by Use of Advanced AmbientGANs

**Fig. 2:**
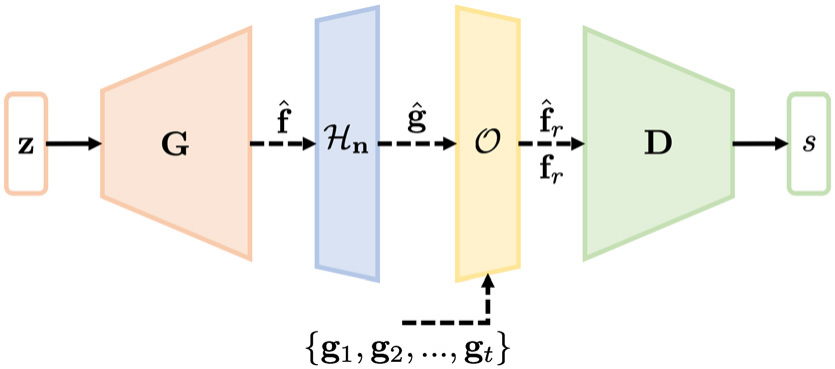
An illustration of the proposed modified AmbientGAN architecture. Any advanced GAN architecture employing a progressively growing training procedure can be employed in this framework.

To train the AmbientGAN using advanced GAN training methods that employ the progressive growing approach, such as ProGAN and Style-based GANs, an image reconstruction operator O: $R^{M}\to R^{N}$ is included in the AmbientGAN architecture. The discriminator is trained to distinguish between the real reconstructed images $f_{r}=O(g)$ and the fake reconstructed images f^r=O(g^). In this way, the generator and the discriminator are associated with images in the same Hilbert space, which enables the use of advanced GAN training methods to train AmbientGANs. This advanced AmbientGAN training strategy is shown in Fig. 2.

Given a training dataset that comprises measured data g, a set of reconstructed images $f_{r}$ is computed by applying the reconstruction operator O to the measured data g. The generator is trained with the discriminator through an adversarial process to generate objects f^=G(z;ΘG) that result in (fake) reconstructed images f^r that are indistinguishable, in distribution, from the real reconstructed images $f_{r}$. This adversarial training process can be represented by a two-player minimax game:

minΘG maxΘD  V(D,G)=Efr∼p(fr)[l(D(fr;ΘD))]+Ef^r∼p(f^r)[l(1−D(f^r;ΘD))], (3)

where f^r=O(ℋn(G(z;ΘG))). As with the original AmbientGAN, when the distribution of objects p(f) uniquely induces the distribution of reconstructed objects $p(f_{r})$, and the generator and the discriminator achieve the global optimum, the trained generator can be employed to produce object samples drawn from the distribution p(f).

**Fig. 3:**
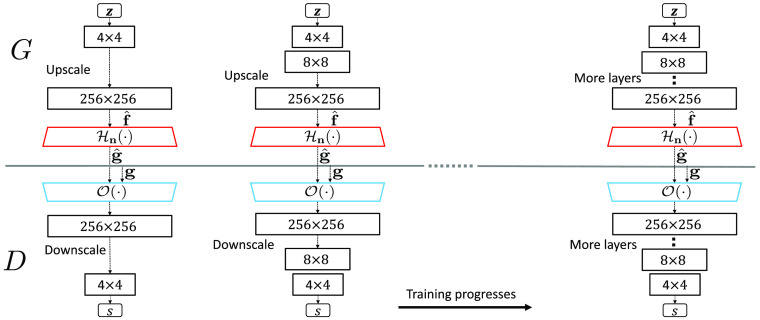
ProAmGAN training procedure. Initially, the generator and discriminator are trained with low-resolution images. Additional layers in the generator and discriminator are trained by use of higher-resolution versions of the original images when the training advances. More details about the progressive growing method can be found in the original ProGAN paper.^33^

It should be noted that when the generator and the discriminator are established progressively using ProGAN or StyleGAN methods, the generator is initially trained to produce low-resolution images that are subsequently upscaled to the original image dimension. The measurement operator $H_{n}$ is subsequently applied to the upscaled images to simulate the measurement data and the reconstructed images are produced using the reconstruction operator O. The reconstructed images are downsampled and the discriminator is subsequently trained on the downsampled (low resolution) reconstructed images. The generator and the discriminator are progressively trained until the original high-resolution images are achieved. The training procedure of AmbientGAN that employs progressive growing strategy is shown in Fig. 3.

**Fig. 4:**
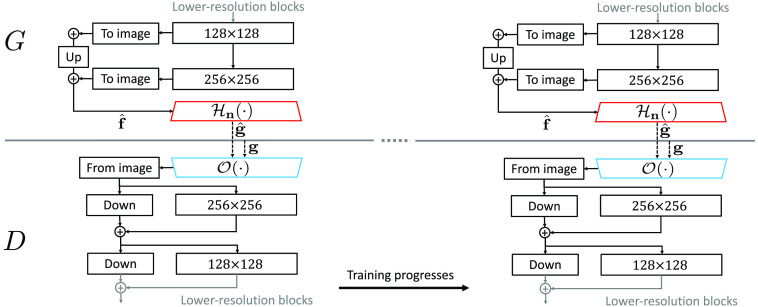
Training procedure of AmbientGAN that employs the StyleGAN2 architecture. The generator employs skip connections and forms the images by explicitly summing images at different resolutions. The discriminator employs residual connections that can be helpful for performing image classification tasks. The “To image” block corresponds to the convolutional operator that maps hidden feature maps having the spatial dimension of n×n (e.g., 128×128) to the grayscale image having the dimension of n×n. Similarly, the “From image” block corresponds to the convolutional operator that maps the grayscale image to the hidden feature maps. The “Down” and “Up” blocks denote bilinear down- and upsampling, respectively. The generator and discriminator are trained without progressive growing (i.e., the complete architectures of the generator and discriminator are trained during the whole training process). More details about the StyeGAN2 architecture can be found in the original StyGAN2 paper.^35^

While the progressive growing strategy has achieved many successes in stabilizing the GAN training for synthesizing high-resolution images, it can cause certain artifacts in the generated images.^35^ As mentioned above, the StyleGAN2 that trains a redesigned generator without progressive growing was developed to further improve the synthesized IQ.^35^ The new generator employs skip connections to form images that are summation of images with different resolutions. This enables the multiresolution training of the generator without the explicit use of progressive growing strategy. The training of AmbientGANs can be potentially improved further by employing the StyleGAN2 generator and discriminator in the proposed AmbientGAN training framework that is shown in Fig. 2. The training procedure of AmbientGAN that employs the StyleGAN2 architecture is shown in Fig. 4.

Below, the advanced AmbientGAN that employs the ProGAN was referred to as ProAmGAN and the one that employs the StyleGAN2 was referred to as Sty2AmGAN.

## 4 Numerical and Experimental Studies

Computer-simulation and experimental studies were conducted to demonstrate the ability of the proposed advanced AmbientGAN methods to establish SOMs from imaging measurements. Details regarding the design of these studies are provided below.

### 4.1 Stylized Imager That Acquires Fully Sampled Data

**Fig. 5:**
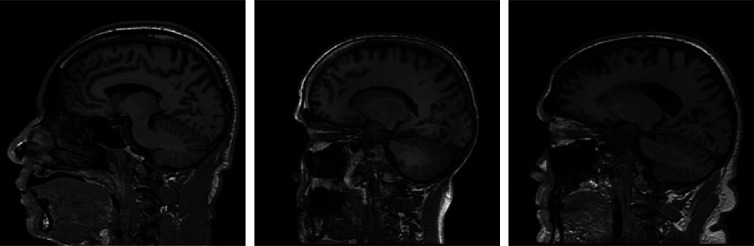
Examples of ground-truth objects f.

A stylized imaging system that acquires fully sampled two-dimensional (2D) Fourier space (a.k.a., k-space) data was investigated first. This imaging system can be described as

g=F(f)+n, (4)

where F denotes a 2D discrete Fourier transform (DFT), f denotes the discretized object to be imaged, and n denotes the measurement noise. While Eq. (4) can be interpreted as a simplified model of MRI, it should be noted that here we do not attempt to model the real-world complexities of data-acquisition in MRI. A situation where modeling error is present is addressed later in Sec. 4.3. A collection of clinical brain MR images from the Alzheimer’s Disease Neuroimaging Initiative (ADNI) database^50^ were employed to serve as ground truth objects f. Fifteen thousand sagittal brain slices of dimension 256×256 were selected from this dataset and were normalized to the range between 0 and 1. These images were employed to form the collection of ground-truth objects f. Examples of f are shown in Fig. 5.

From the ensemble of objects f, k-space measurement data were simulated according to Eq. (4). The measurement noise n was modeled by i.i.d. zero mean complex Gaussian distribution with a standard deviation of $\sigma_{n}(g)$ for both the real and imaginary components. Different measurement noise levels corresponding to standard deviations $\sigma_{n}(g)=4$ and 16 were considered.

From each ensemble of simulated k-space data, reconstructed images $f_{r}$ were produced by acting a 2D inverse discrete Fourier transform (IDFT) operator $F^{-1}$ to the measured image data g and taking the real component: $f_{r}=\text{Re}(F^{-1}(g))$. For each noise level, the proposed AmbientGANs were trained to establish an SOM that characterizes the distribution of objects f using the ensemble of reconstructed noisy images $f_{r}$. For comparison, standard (i.e., nonambient) ProGANs were trained directly using the reconstructed images $f_{r}$. In this case, because the reconstructed images are affected by measurement noise, the resulting generator will learn to sample from the distribution of noisy reconstructed images instead of the distribution of (noiseless) objects f.

The Fréchet inception distance (FID)^51^ score, a widely employed metric for assessing generative models, was computed to evaluate the performance of the original ProGAN and the proposed AmbientGANs. The FID score quantifies the distance between the features extracted by the Inception-v3 network^52^ from the ground-truth (real) and generated (fake) objects. Lower FID score indicates better quality and diversity of the generated objects. The FID scores were computed using 15,000 ground-truth objects, 15,000 ProGAN-generated objects, 15,000 ProAmGAN-generated objects, and 15,000 Sty2AmGAN-generated objects.

As another form of comparison between the ProGAN- and AmbientGANs-generated images, the standard deviation of the noise in the generated images σn(f^) was estimated. Specifically, a previously described method^53^ was applied to 15,000 ProGAN-generated images, 15,000 ProAmGAN-generated images, and 15,000 Sty2AmGAN-generated images. The average of the estimated standard deviation of the noise in the ProGAN- and AmbientGANs-generated images was compared.

### 4.2 Stylized Imager That Acquires Incomplete Data

Imaging systems sometimes acquire undersampled, or incomplete, measurement data to accelerate the data-acquisition process or for other purposes. In such cases, the imaging operator H has a nontrivial null space and only the measurement component $f_{\mathrm{meas}}=H^{†}\mathrm{Hf}$ can be observed by the imaging system, where $H^{†}$ denotes the Moore–Penrose pseudoinverse of H. Because of this, it is expected that the performance of an AmbientGAN trained using incomplete measurements will be adversely affected by this information loss. This topic is investigated below and the extent to which ProAmGANs and Sty2AmGANs can learn to sample from the distribution of measurement components of an object is demonstrated.

The ensemble of 15,000 clinical MR images that was described in Sec. 4.1 was employed to serve as ground truth objects. Three accelerated data-acquisition designs that undersample k-space by use of the Cartesian sampling pattern with an acceleration factor (also known as the reduction factor) R of 1.25, 2, and 4 were considered. The acceleration factor R is defined as the ratio of the amount of fully sampled k-space data to the amount of k-space data collected in the accelerated data-acquisition process. For each considered design, a collection of 15,000 measured data g were simulated by computing and sampling the k-space data and adding i.i.d. zero mean Gaussian noise with a standard deviation of 4 to both the real and imaginary components.

A stylized imager was considered in which undersampled k-space data were acquired and $H^{†}$ could therefore be computed by applying a 2D IDFT to the zero-filled k-space data. For each data-acquisition design, reconstructed objects $f_{r}$ were produced by acting $H^{†}$ on the given measured image data g. A ProAmGAN and a Sty2AmGAN were subsequently trained to establish an SOM for each data-acquisition design. In the training process, H and $H^{†}$ were applied to the generator-produced objects as discussed in Sec. 3. The FID scores were computed by use of 15,000 ground-truth objects f and 15,000 AmbientGANs-generated objects f^ for each data-acquisition design. To assess the ability of ProAmGANs and Sty2AmGANs to accurately learn the variation in the measurement components of the objects, the FID score was computed by use of the ground-truth measurement components $f_{\mathrm{meas}}=H^{†}\mathrm{Hf}$ and AmbientGANs-generated measurement components f^meas=H†Hf^ for each data-acquisition design.

### 4.3 Experimental Emulated Single-Coil MRI Data

As a step toward transcending the stylized studies, an emulated set of single-coil knee MRI k-space measurements were also employed to train a ProAmGAN and Sty2AmGAN. These measurements were obtained from the NYU fastMRI Initiative database.^54^ The central 256×256 regions of the k-space were extracted and a total of 11,400 k-space acquisitions were employed for model training. The reconstructed images were formed as the magnitude of the IDFT of the k-space data. The magnitude MR images are commonly employed in MRI because they can avoid the phase artifacts that are commonly present in experimental MR measurement data.^55^

When training the ProAmGAN and Sty2AmGAN, the canonical measurement model was assumed: g^=Hn(f^)=F(f^)+n. However, when dealing with experimental measurements, the noise model that characterizes n is unknown and needs to be estimated. This was accomplished as follows. The noise in the (emulated) experimental k-space measurements was assumed to be described by i.i.d. complex-valued Gaussian random variables; accordingly, the noise in the reconstructed magnitude MR image was modeled by a Rayleigh distribution.^55^ The standard deviation of the measurement noise was subsequently estimated by fitting a Rayleigh distribution to a set of patches, residing outside the support of the object, in the magnitude images that were reconstructed from the noisy k-space measurements. The estimated standard deviation specified the k-space noise model in the measurement model above.

To train the ProAmGAN and Sty2AmGAN by employing the magnitude MR images as the input to the discriminator, care must be taken when computing the simulated reconstructed image f^r. Specifically, if the modulus operator, which is denoted as abs(·), is directly applied to the IDFT of the simulated k-space measurements g^, i.e., f^r=abs(F−1(g^))≡abs(f^+F−1(n)), the fake magnitude images f^r can be indistinguishable from the real magnitude images $f_{r}$ despite the fact that the corresponding fake objects f^ can be negative. This can prevent the generator from being properly trained for use as an SOM.

To address this issue, we computed the fake reconstructed image f^r as

f^r=f^+e, (5)

where e=abs(F−1(F(ReLU(f^))+n))−ReLU(f^). Here, ReLU(·) is the componentwise rectified linear unit (ReLU) operator that outputs the input value if the input value is positive; while if the input value is negative, it outputs 0. The quantity f^r can be subsequently expressed as

f^r={abs(F−1(F(f^)+n)),if  f^≥0f^+abs(F−1(n)),if  f^<0. (6)

In this way, fake reconstructed images f^r that are produced by positive objects can represent the corresponding magnitude images while those that are produced by negative objects cannot represent magnitude images. Therefore, when the training is completed such that the fake reconstructed images f^r are indistinguishable from the ground-truth magnitude MR images $f_{r}$, the generator would produce non-negative objects.

### 4.4 Task-Based Image Quality Assessment

**Fig. 6:**
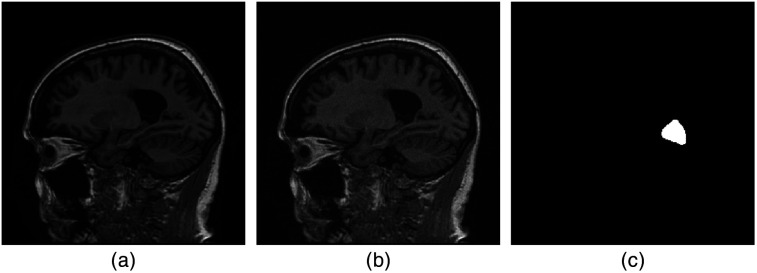
(a) An example of a ground truth, or “real,” object f, (b) the corresponding noisy signal-absent image $g_{t}$, and (c) the considered signal s.

The generative models established using the ProGANs, ProAmGANs, and Sty2AmGANs in the stylized numerical studies described in Secs. 4.1 and 4.2 were further evaluated using objective measures of IQ. To accomplish this, a signal-known-exactly and background-known-statistically binary classification task was considered. A Hotelling observer (HO) was employed to classify noisy images $g_{t}$ as satisfying either a signal-absent hypothesis ($H_{0}$) or signal-present hypothesis ($H_{1}$):

$$H_{0}:\;g_{t}=f+n_{t},$$ (7a)

$$H_{1}:\;g_{t}=f+s+n_{t},$$ (7b)

where s denotes the considered signal placed at the fixed location and $n_{t}$ is i.i.d. zero-mean Gaussian noise having the standard deviation of 2%. Two studies were conducted in which the background objects f corresponded to ground truth brain images or synthetic images produced using an AmbientGAN. As such, this study sought to determine whether the GAN-generated objects could “fool” the HO on the specified detection task. An example of the “real” object f, the corresponding noisy signal-absent image $g_{t}$, and the considered signal are shown in Fig. 6.

The considered signal detection task was performed on a region of interest (ROI) of dimension of 64×64  pixels centered at the signal location. The signal-to-noise ratio of the HO test statistic ${\mathrm{SNR}}_{\mathrm{HO}}$ was employed as the figure-of-merit for assessing the IQ:^7^

$${\mathrm{SNR}}_{\mathrm{HO}}=\sqrt{s_{\mathrm{ROI}}^{T}K^{-1}s_{\mathrm{ROI}}},$$ (8)

where $s_{\mathrm{ROI}}\in R^{4096\times 1}$ denotes the vectorized signal image in the ROI, and $K\in R^{4096\times 4096}$ denotes the covariance matrix corresponding to the ROIs in the noisy MR images. When computing ${\mathrm{SNR}}_{\mathrm{HO}}$, $K^{-1}$ was calculated using a covariance matrix decomposition.^7^ The values of ${\mathrm{SNR}}_{\mathrm{HO}}$ were computed by use of 15,000 generated objects produced by each trained generative model. They were compared to the ${\mathrm{SNR}}_{\mathrm{HO}}$ computed by use of 15,000 ground truth objects.

### 4.5 Training Details

All ProGAN, ProAmGAN, and Sty2AmGAN models were trained by use of Tensorflow^56^ on 2 NVIDIA Quadro RTX 8000 GPUs. The Adam algorithm,^57^ which is a stochastic gradient algorithm, was employed as the optimizer in the training process. To implement the ProAmGAN, the ProGAN code; available in a Github repository: https://github.com/tkarras/progressive_growing_of_gans, was modified according to Fig. 2. Specifically, for each considered imaging system, the corresponding measurement operator was applied to the generator-produced images for simulating the measurement data and the reconstruction operator was applied to the measurement data for producing the reconstructed images used as the input to the discriminator. The default ProGAN architecture with the latent space having the dimensionality of 512 and the initial image resolution of 4×4 was employed to implement the ProAmGANs for the considered numerical studies. Additional details about the ProGAN architecture and the progressive growing training method can be found in the literature.^33^

The Sty2AmGAN was implemented by modifying the StyleGAN2 code (available in a Github repository: https://github.com/NVlabs/stylegan2) by augmenting the StyleGAN2 with the measurement operator $H_{n}$ and the reconstruction operator O according to Fig. 2. For the considered experimental study, the default StyleGAN2 architecture (i.e., “config F” ^35^) with the input latent space having the dimensionality of 512 was employed to implement the Sty2AmGAN. Additional details regarding the StyleGAN2 architecture and the corresponding training strategy can be found in the literature.^35^

During the training of ProAmGANs and Sty2AmGANs, the latent vectors were sampled from the standard normal distribution to generate fake images. We visually examined these generated fake images and stopped the training after these images possessed a plausible visual quality or did not visually improve significantly. We acknowledge that this stopping rule is subjective, and it remains an open problem to quantitatively evaluate AmbientGANs to be applied in situations where ground-truth objects are not accessible.

## 5 Results

### 5.1 Stylized Imager That Acquires Fully Sampled Data

**Fig. 7:**
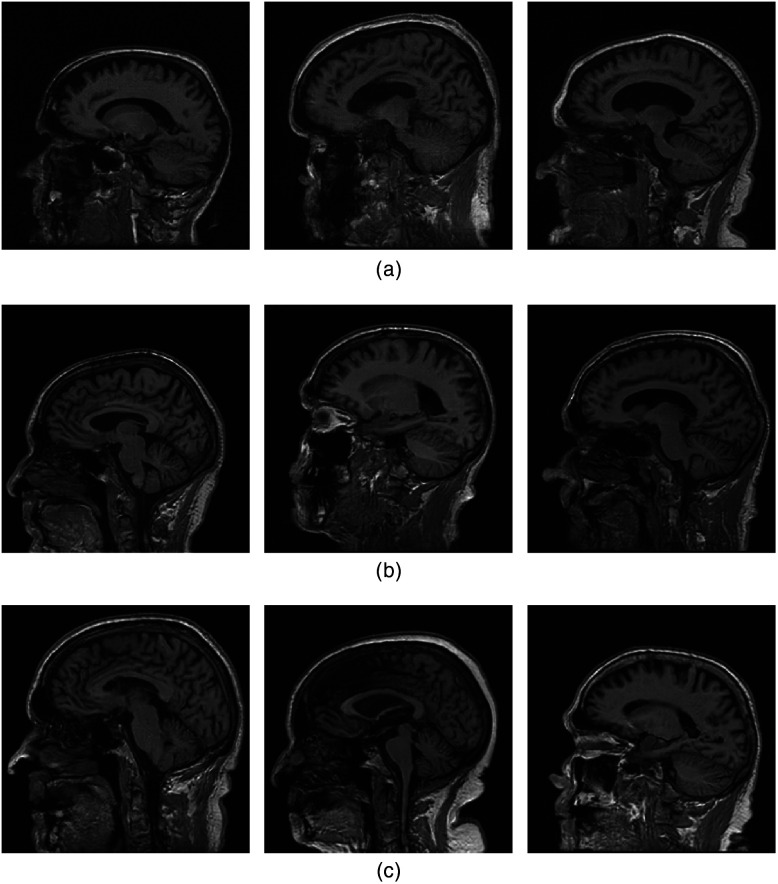
ProGAN-generated (top row), ProAmGAN-generated (middle row), and Sty2AmGAN-generated (bottom row) images corresponding to $\sigma_{n}(g)=4$.

**Fig. 8:**
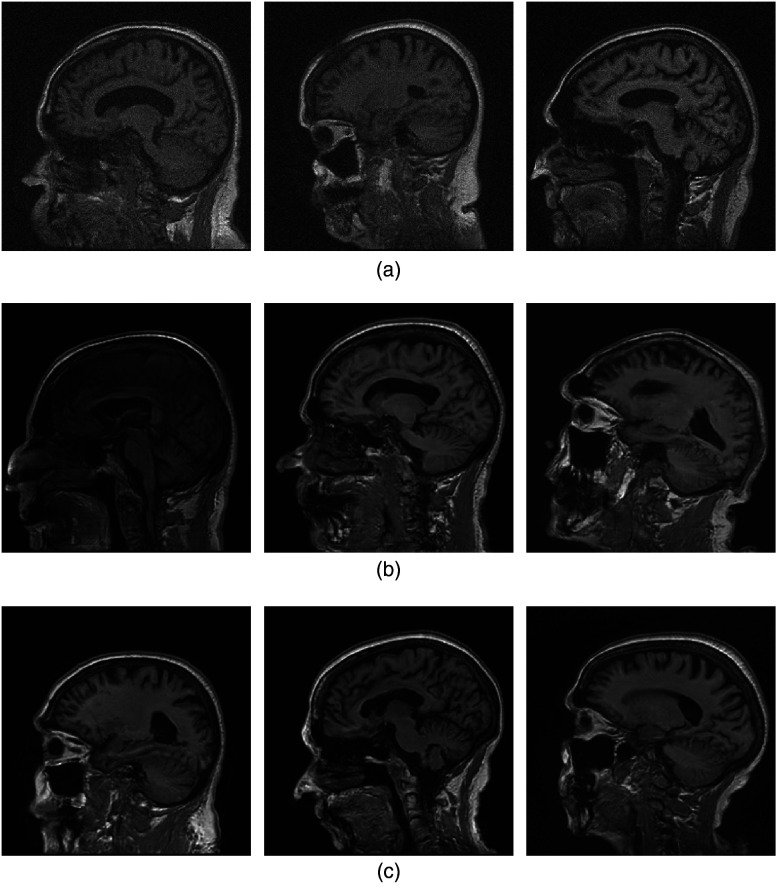
ProGAN-generated (top row), ProAmGAN-generated (middle row), and Sty2AmGAN-generated (bottom row) images corresponding to $\sigma_{n}(g)=16$.

Images that were synthesized by use of the advanced-AmbientGANs and ProGANs that were trained using fully sampled noisy k-space data or images reconstructed from them, respectively, are shown in Figs. 7 and 8. These correspond to measurement noise levels of 4 and 16, respectively.

It is observed that the ProGAN-generated images contain significant noise when $\sigma_{n}(g)=16$, while the ProAmGAN and Sty2AmGAN generated clean images that do not contain significant noise. This demonstrates the ability of the ProAmGAN and Sty2AmGAN to mitigate measurement noise when establishing SOMs.

**Fig. 9:**
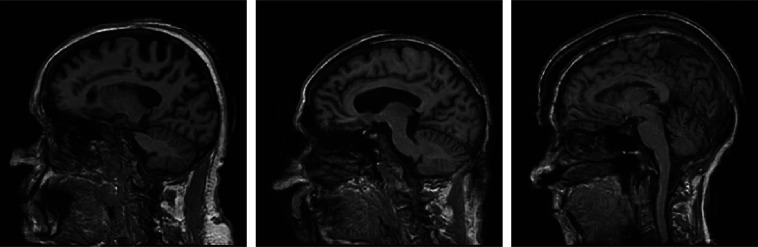
ProAmGAN-produced images having significant artifacts near the skulls that were not observed in Sty2AmGAN-produced images. These images were produced by the ProAmGAN corresponding to $\sigma_{n}(g)=4$.

The FID scores, estimated standard deviation of the noise in the generated images σn(f^), and ${\mathrm{SNR}}_{\mathrm{HO}}$ were evaluated for the ProGANs, ProAmGANs, and Sty2AmGANs. These metrics are shown in Table 1. The ProAmGANs produced FID scores that were smaller than those produced by the ProGANs, which indicates that the ProAmGANs outperformed the ProGANs. In addition, Sty2AmGANs can further improve the synthesized IQ and produced FID scores smaller than the ProAmGANs. It was also observed that ProAmGANs can produce images having artifacts that did not appear in Sty2AmGAN-produced images. Examples of such images are shown in Fig. 9. The estimated standard deviation of the noise in the ProGAN-generated images increased nearly linearly as the standard deviation of measurement noise was increased; while the estimated standard deviation of the noise in the ProAmGAN and Sty2AmGAN-generated images were almost unchanged. The ${\mathrm{SNR}}_{\mathrm{HO}}$ values corresponding to the ProGANs had negative biases to the reference value that were computed by use of ground-truth objects, and this negative bias became more significant as the measurement noise level increased. This is because the ProGANs capture both the object variability and the noise randomness, instead of object variability alone, which degrades the estimated observer performance. The ${\mathrm{SNR}}_{\mathrm{HO}}$ values corresponding to the ProAmGANs and Sty2AmGANs were closer to the reference value.

**Table 1:** The FID score of the objects, the estimated noise standard deviation, and the ${\mathrm{SNR}}_{\mathrm{HO}}$ (the reference value 1.72) corresponding to the objects produced by the ProGANs, ProAmGANs, and Sty2AmGANs that were trained with fully sampled noisy k-space measurement data.

	ProGAN		ProAmGAN		Sty2AmGAN	
	$\sigma_{n}(g)=4$	$\sigma_{n}(g)=16$	$\sigma_{n}(g)=4$	$\sigma_{n}(g)=16$	$\sigma_{n}(g)=4$	$\sigma_{n}(g)=16$
FID (f^)	25.31	146.92	17.60	37.66	13.84	21.24
σn(f^)	1.77%	6.43%	0.59%	0.51%	0.59%	0.59%
${\mathrm{SNR}}_{\mathrm{HO}}$	1.62	1.10	1.73	1.78	1.70	1.66

### 5.2 Stylized Imager That Acquires Incomplete Data

**Fig. 10:**
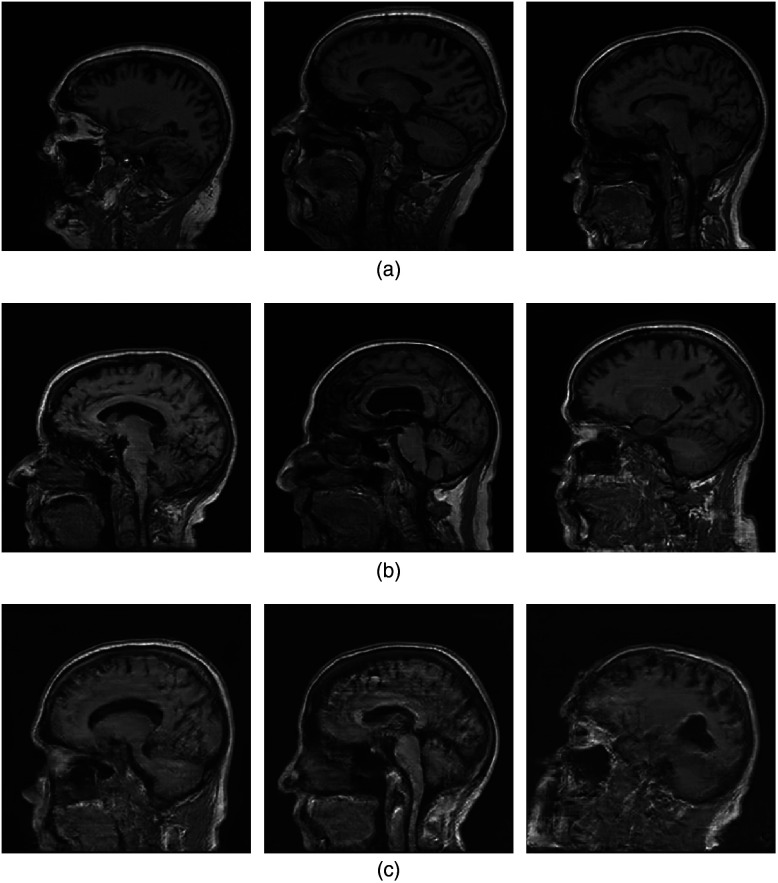
(a) ProAmGAN-generated images corresponding to the k-space sampling acceleration factor R of 1.25. (b) The corresponding images for R=2. (c) The corresponding images for R=4.

**Fig. 11:**
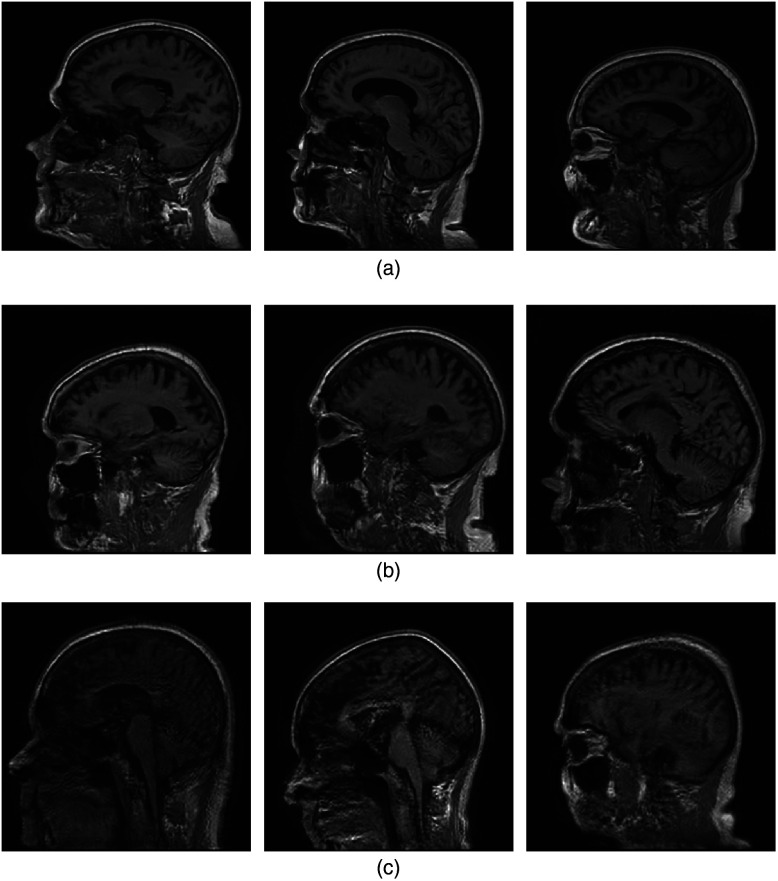
(a) Sty2AmGAN-generated images corresponding to the k-space sampling acceleration factor R of 1.25. (b) The corresponding images for R=2. (c) The corresponding images for R=4.

Images that were synthesized using the ProAmGANs and Sty2AmGANs that were trained using undersampled k-space measurement data acquired with different acceleration factors are shown in Figs. 10 and 11, respectively. The images produced by the ProAmGANs and Sty2AmGANs corresponding to the acceleration factor R=1.25 and 2 are visually plausible; while when the acceleration factor was increased to 4, the generated-images were obviously contaminated by artifacts and some structures were distorted. This demonstrates that the ProAmGAN and Sty2AmGAN were adversely affected by the incompleteness of the measurement data acquired by imaging systems having nontrivial null-space.

The quantitative metrics that include FID scores and ${\mathrm{SNR}}_{\mathrm{HO}}$ are summarized in Table 2. The FID scores produced by the Sty2AmGANs were smaller than those produced by the ProAmGANs. This indicates that the Sty2AmGANs outperformed the ProAmGANs in terms of FID scores. For both the ProAmGANs and Sty2AmGANs, the FID scores corresponding to the generated objects f^ were increased when the acceleration factor R increased, which indicates that the ProAmGAN and Sty2AmGAN were detrimentally affected by the null space of the imaging operator. However, the FID scores corresponding to the measurement components of the generated objects f^meas were not significantly affected, which suggests that the ProAmGAN and Sty2AmGAN can reliably learn the distribution of the measurement components of the objects. The ${\mathrm{SNR}}_{\mathrm{HO}}$ values produced by the ProAmGANs and Sty2AmGANs increased when the k-space sampling acceleration factor R increased. This suggests that the ability of AmbientGANs to learn object variation that limits observer performance can be decreased when the null space of the imaging operator becomes large.

**Table 2:** The FID score of the objects, the FID score of the measurement components, and the ${\mathrm{SNR}}_{\mathrm{HO}}$ (reference value 1.72) corresponding to the objects produced by the ProAmGANs and Sty2AmGANs that were trained with undersampled k-space data with different acceleration factors.

	ProAmGAN			Sty2AmGAN		
	R=1.25	R=2	R=4	R=1.25	R=2	R=4
FID (f^)	20.64	39.25	118.27	16.40	37.76	109.41
FID (f^meas)	12.83	13.25	8.96	10.52	12.51	11.80
${\mathrm{SNR}}_{\mathrm{HO}}$	1.75	1.80	1.84	1.66	1.73	1.77

### 5.3 Experimental Emulated Single-Coil MRI Data

**Fig. 12:**
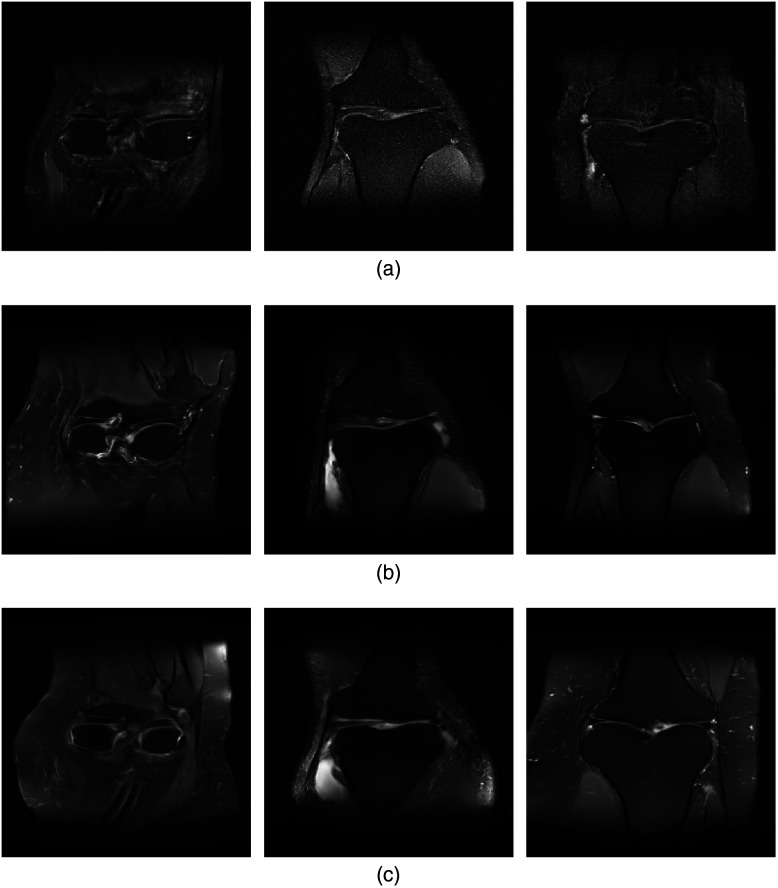
Results from emulated single-coil MRI data. (a) ProGAN-generated images. (b) ProAmGAN-generated images. (c) Sty2AmGAN-generated images.

**Fig. 13:**
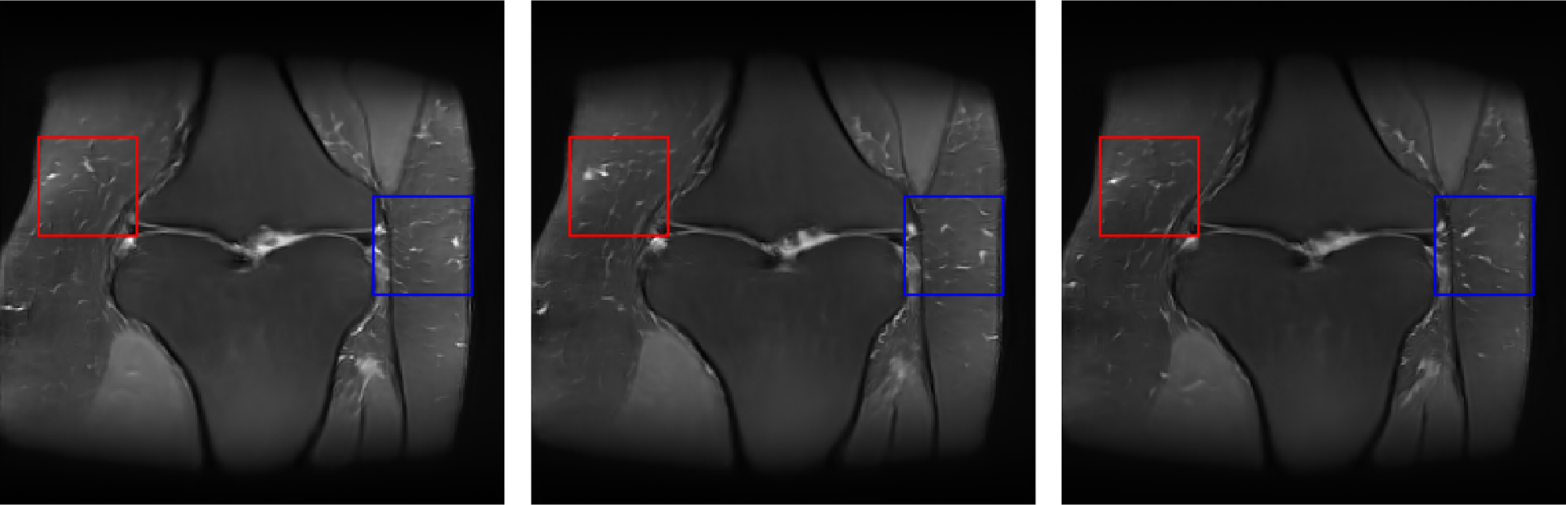
Sty2AmGAN-generated objects having similar large-scale structures but different fine-scale subcutaneous fat textures. Textures in the red and blue rectangles have different appearances.

Images produced by the ProGAN, ProAmGAN, and Sty2AmGAN are shown in the top row, middle row, and bottom row of Fig. 12, respectively. The ProGAN-produced images were contaminated by noise because the ProGAN was trained directly by use of noisy reconstructed images. Both the ProAmGAN and Sty2AmGAN produced images that did not appear to be degraded by noise, which demonstrates the ability of advanced AmbientGAN strategies to mitigate the measurement noise when establishing an SOM. The Sty2AmGAN can further improve the training of the AmbientGAN for establishing the SOM. For example, the images produced by the ProAmGAN were more blurred than those produced by the Sty2AmGAN. Because the ground-truth objects corresponding to the synthesized images were not accessible in this experimental study, only a subjective visual assessment was performed. The style-based generator used in Sty2AmGAN can provide additional ability to control scale-specific image features.^58^^,^^59^ To demonstrate this, as shown in Fig. 13, we controlled the style-based generator of the Sty2AmGAN to produce knee images having similar large-scale structures but different fine-scale subcutaneous fat textures. These images were produced by use of the same latent vector but different latent noise maps that are extra inputs to the style-based generator. More details about the latent noise maps and the scale-specific manipulation of the style-based generators can be found in the references.^34^^,^^35^

## 6 Discussion and Conclusion

It is known that it is important to address object variability when computing objective measures of IQ for use in imaging system characterization or optimization. When computer-simulation studies are employed, SOMs are the means by which this can be accomplished. However, establishing realistic SOMs from experimental image data has remained challenging and few methods are available to accomplish this.

Motivated by this need, a methodology for training AmbientGANs by use of medical image data was proposed in this study. The trained generator of the AmbientGAN represents the sought-after SOM. The proposed methodology enables the use of advanced GAN training methods and architectures in the AmbientGAN training and therefore permits the AmbientGAN to be applied to realistically sized medical image data. To demonstrate this, two specific advanced AmbientGANs were considered: ProAmGANs and Sty2AmGANs.

Stylized numerical studies were systematically conducted in which Sty2AmGANs and ProAmGANs were trained on simulated measurement data corresponding to an object ensemble and a variety of stylized imaging systems. Both visual examinations and quantitative analyses, including task-specific validations, demonstrated that the proposed ProAmGANs and Sty2AmGANs hold promise to establish realistic SOMs from imaging measurements. In addition, an experimental study was conducted in which the ProAmGAN and Sty2AmGAN were trained on emulated experimental MRI measurement data. This demonstrated the effectiveness of the methods under less stylized conditions in which modeling error was present.

In addition to objectively assessing imaging systems and data-acquisition designs, the SOMs established by the proposed advanced AmbientGAN methods can be employed to regularize image reconstruction problems. Recent methods have been developed for regularizing image reconstruction problems based on GANs such as compressed sensing using generative models^60^ and image-adaptive GAN-based reconstruction methods.^61^^,^^62^ Sty2AmGANs can also be used for prior image-constrained reconstruction.^59^ Furthermore, the established SOMs may be employed to produce clean reference images for training deep neural networks for solving other image-processing problems such as image denoising^63^^,^^64^ and image super-resolution.^65^ However, it should be noted that the AmbientGANs-established SOMs were not uninfluenced by the imaging systems. As shown in the numerical studies, the IQ of the AmbientGAN-generated images was affected to varying extents when different levels of measurement noise and different degrees of incompleteness of the measurement data were considered. In addition, the generated images can be contaminated by artifacts and distorted structures. The artifacts generated by AmbientGANs and the impact of imaging systems on the training of AmbientGANs may limit the use of the proposed AmbientGANs in certain medical imaging applications. It will be important to investigate the extent to which the AmbientGANs can be successfully applied for solving specific medical imaging problems.

There remain additional topics for future investigation. We have conducted a preliminary objective assessment of the AmbientGANs by use of the HO^7^^,^^66^ and a binary signal detection task. In this preliminary study, the generated images were objectively assessed by use of binary signal detection studies. It will be important to validate the SOMs established by use of the proposed methods when clinically relevant tasks are addressed by a variety of numerical observers such as the ideal observer that deploys higher-order statistical information of images^67^^–^^70^ and anthropomorphic observers that mimic human performance.^71^ Moreover, it will also be important to validate the AmbientGANs-established SOMs by use of other image features such as variations of certain textures and shape distributions of different organ structures of the synthesized objects. To implement the proposed AmbientGAN methods, the imaging forward operator needs to be accurately modeled. In addition, in practice, medical imaging measurement data are frequently acquired under inhomogeneous imaging conditions. It will be important to investigate the impact of the modeling error of the imaging forward operator and the inhomogeneous imaging conditions on the ability of AmbientGANs to establish SOMs for certain observers and tasks.
